# Chemical composition and hepatoprotective effect of essential oil from *Myrtus communis* L. flowers against CCL_4_-induced acute hepatotoxicity in rats

**DOI:** 10.1039/c8ra08204a

**Published:** 2019-01-28

**Authors:** Anis Ben Hsouna, Sabah Dhibi, Wissal Dhifi, Wissem Mnif, hmed Ben Nasr, Najla Hfaiedh

**Affiliations:** Department of Life Sciences, Faculty of Sciences of Gafsa Zarroug 2112 Gafsa Tunisia benhsounanis@yahoo.fr; Laboratory of Biotechnology and Plant Improvement, Centre of Biotechnology of Sfax Tunisia; Unit of Macromolecular Biochemistry and Genetics, Faculty of Sciences of Gafsa Sidi Ahmed Zarrouk 2112 Gafsa Tunisia sabahdhibi7@gmail.com hfaiedhnajla@gmail.com; LR17-ES03 Physiopathology, Food and Biomolecules, Higher Institute of Biotechnology of Sidi Thabet, Biotechpole Sidi Thabet 2020 Ariana Tunisia wissal_d2002@yahoo.fr; Faculty of Sciences and Arts in Balgarn, University of Bisha PO Box 60 Balgarn-Sabt Al Olaya 61985 Kingdom of Saudi Arabia w_mnif@yahoo.fr; University of Manouba, ISBST, BVBGR-LR11ES31, Biotechpole Sidi Thabet 2020 Ariana Tunisia

## Abstract

*Myrtus communis* L. (Myrtle) is one of the most important aromatic and medicinal species from the Myrtaceae family. It is traditionally used as antiseptic, disinfectant drug and hypoglycemic agent. The aim of our study was to evaluate the protective effect of *Myrtus communis* essential oil (*Mc*EO) on CCl_4_-induced hepatotoxicity in rat. Thirty two adult Wistar rats were divided into 4 groups of 8 each: (1) a control group; (2) was given a single dose of CCl_4_ (1 mL kg^−1^ in 1% olive oil. ip) on the 14^th^ day (3) were given during 15 days a daily i.p. injection of *Mc*EO at 250 mL kg^−1^ b.w (4) a group was pretreated with *Mc*EO and intoxicated with CCl_4_ on the 14^th^ day. The major components of *Mc*EO are α-pinene (35.20%), 1,8-cineole (17%), linalool (6.17%) and limonene (8.94%) which accounted for 67.31% of the whole oil. The antioxidant activity of *Mc*EO was evaluated using DPPH scavenging ability, β-carotene bleaching inhibition and hydroxyl radical-scavenging activity. Moreover, the effect of *Mc*EO (250 mg kg^−1^ body weight BW) administrated for 14 consecutive days was evaluated in wistar rat. Administration of a single dose of CCl_4_ caused hepatotoxicity as monitored by an increase in lipid peroxidation (thiobarbituric acid reactive substances) as well in protein carbonyl level but decreased in antioxidant markers in the liver tissue. The *Mc*EO pre-treatment significantly prevented the increased plasma levels of hepatic markers and lipid levels induced by CCl_4_ in rats. Furthermore, this fraction improved biochemical and histological parameters as compared to CCl_4_-treated group. Our results suggest that *M. communis* contains promising substances to counteract the CCl_4_ intoxication and which may be efficient in the prevention of hepatotoxicity complications.

## Abstract 2

*Myrtus communis* L. (Myrtle) is one of the most important aromatic and medicinal species from the Myrtaceae family.

## Graphical abstract

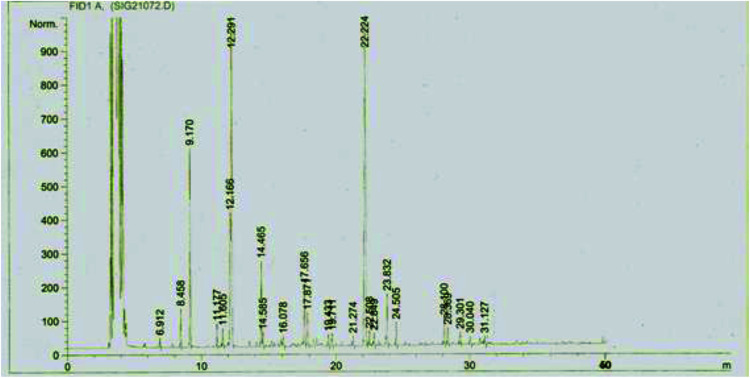

## Introduction

Because it is the major site of detoxification and xenobiotic metabolism, the liver is usually injured by toxic chemicals, drugs, and infiltrated virus and bacteria by ingestion or infection.^1^ To investigate hepatotoxicity and understand its mechanisms and eventually to test several treatments carbon tetrachloride (CCl_4_) has been extensively used to induce liver injury in preclinical animal studies^2^ CCl_4_ is considered as one of the best characterized compounds to induce human cirrhosis like effects in experimental animals. The current options for the treatment of liver diseases include pharmacotherapy, surgery, and liver transplantation. All of these treatments have shown limited therapeutic benefits and are associated with serious complications. It is worth noticing that the treatment with steroids, vaccines, and antiviral drugs is not only efficient but also associated with serious risks of toxicity, especially if administered chronically or sub-chronically.^3^ Obviously, there is a critical need for exploring novel and alternative approaches for to wound heal liver diseases.^4,5^ In the absence of a reliable liver protective drug in the modern system of medicine, a number of medicinal plants are recommended for the treatment of liver disorders to evaluate the efficacy of hepato-protectants.^6^ In this context, medicinal plants are a source of a large number of bioactive compounds which could be exploited in drug development program for the treatment of many diseases among them liver injury. The protective role of plants is particularly due to their antioxidative constituents which are able to delay or inhibit the reactive oxygen species generation.^7–9^

Many studies proved that plant extracts are very rich in antioxidant compounds that offered an effective protection against CCl_4_ induced hepatotoxicity by inhibiting lipid peroxidation and enhancing antioxidant enzyme activity.^9,10^

Among medicinal plants endemic to Tunisia, *Myrtus communis* L. (Myrtle) is one of the most important aromatic and medicinal species belonging to the Myrtaceae family. It is traditionally used as an antiseptic, disinfectant, anti inflammatory and hypoglycemic agent.^11^ This plant is also used as flavor in food and cosmetic industries.^12^ Up to date, the majority of studies of myrtle have focused on its leaf and berries volatile fraction and phenolic compounds.^13^ In this study, we investigated the antioxidant effects of the *Mc*EO and its hepato protective effects against CCl_4_ induced liver injury.

## Materials and methods

*M. communis* flowers were collected from El Kef locality (Tunisia, 35.23° N, and 11.11° E), in June 2016. Plant identification was carried out by Pr. Ferjani Ben Abduallah (Faculty of Science of Sfax, Tunisia).

### Essential oil extraction

The essential oils (EOs) have been extracted from one kilogram air-dried flowers separately by hydrodistillation for 3 h, using a Clevenger-type apparatus. The aqueous phase was extracted with dichloromethane (3 × 50 mL) and dried with anhydrous sodium sulphate. After filtration, the solvent is eliminated by pressure distillation reduced in a rotary evaporator and pure oil was stored at 4 °C in obscurity till the beginning of *Mc*EO analysis.^14^ Essential oil yields were estimated on the basis of the dry weight of plant material as: *Mc*EO (% v/w) = observed volume of oil (mL)/weight of sample (g) × 100.

### Gas chromatography-mass spectrometry (GC/MS)

The analysis of the *Mc*EO was performed according to GC/MS HP model 6980 inert MSD (Agilent Technologies, J&W Scientific Products, Palo Alto, CA, USA), equipped with an Agilent Technologies capillary HP-5MS column (60 m length; 0.25 mm i.d; 0.25 mm film thickness), and coupled to a mass selective detector (MSD5973, ionization voltage 70 eV; all Agilent, Santa Clara, CA). The carrier gas was helium and was used at 1.2 mL min^−1^ flow rate. The oven temperature program was as follows: 1 min at 100 °C ramped from 100 to 280 °C at 5 °C min^−1^ and 25 min at 280 °C. The chromatograph was equipped with a split/splitless injector used in the splitless mode. Identification of components was assigned by matching their mass spectra with Wiley Registry of Mass Spectral Data 7^th^ edition (Agilent Technologies, Inc.) and National Institute of Standards and Technology 05 MS (NIST) library data.^14^

### Antioxidant testing assays

#### DPPH radical scavenging activity

Radical scavenging activity of *Mc*EO was determined using 1-diphenyl-2-picrylhydrazyl (DPPH) radical as a reagent according to the method of Hatano *et al.*^15^ with some modifications. Briefly, 1 mL of a 4% (w/v) solution of DPPH radical in ethanol was mixed with 500 μL of sample solutions (different concentrations). The mixture was incubated for 20 min in the dark at room temperature. Scavenging capacity was read spectrophotometrically by monitoring the decrease of the absorbance at 517 nm. Lower absorbance of the reaction mixture indicates higher free radical scavenging activity. Ascorbic acid was used as standard. The percent DPPH scavenging effect was calculated using the following equation: DPPH scavenging effect (%) = (*A*_control_ − *A*_sample_/*A*_control_) × 100. Where *A*_control_ is the absorbance of the control reaction were the sample is replaced by 500 μL ethanol. Tests were carried out in triplicate.

#### β-Carotene bleaching assay

The antioxidant activity was determined according to the β-carotene bleaching method described by Koleva *et al.*^16^ A stock solution of β-carotene–linoleic acid mixture was prepared as follows: 0.5 mg of β-carotene was dissolved in 1 mL of chloroform with 25 μL of linoleic acid and 200 mg of Tween-20. Chloroform was completely evaporated, using a vacuum evaporator. Then, 100 mL of distilled water, saturated with oxygen (30 min), were added and the obtained solution was vigorously shaken. A 4 mL of this reaction mixture were dispensed into test tubes and 200 μL of each sample, prepared at different concentrations, were added. The emulsion system was incubated for 2 h at 50 °C. The same procedure was repeated with Butylatedhydroxytoluene (BHT) as positive control, and a blank as a negative control. After this incubation period, the absorbance of each mixture was measured at 490 nm. Antioxidant activity in β-carotene bleaching model in percentage (*A*%) was calculated with the following equation: 
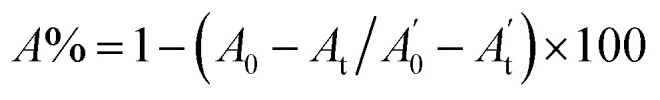
, where *A*_0_ and 
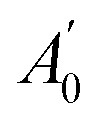
 are absorbances of the sample and the blank, respectively, measured at zero time, and *A*_t_ and 
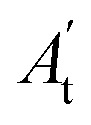
 are absorbances of the sample and the blank, respectively, measured after 2 h. All tests were carried out in triplicate.

#### Hydroxyl radical-scavenging activity

The hydroxyl radical scavenging activity was determined according to the colorimetric deoxyribose oxidation by the Fenton reaction leading to malondialdehyde.^17^ The hydroxyl radicals were generated from the Fe^3+^/ascorbate/EDTA/H_2_O_2_ system in the non site-specific assay or Fe^3+^/ascorbate/H_2_O_2_ in the site-specific assay. The reacting mixture for the deoxyribose assay contained in a final volume of 1 mL the following reagents: 200 μL KH_2_PO_4_–KOH (100 mM), 200 μL deoxyribose (15 mM), 200 μL FeCl_3_ (500 μM), 100 μL EDTA (1 mM), 100 μL ascorbic acid (1 mM), 100 μL H_2_O_2_ (10 mM) and 100 μL sample of different concentration of essential oil (10–80 μg mL^−1^). The reaction mixtures were incubated at 37 °C for 1 h. At the end of the incubation period, 1 mL of 1% (w/v) TBA was added to each mixture followed by the addition of 1 mL of 2.8% (w/v) TCA. The solutions were heated in a water bath at 80 °C for 20 min to develop the pink coloured malondialdehyde–thiobarbituric acid: MDA–TBA adduct, and the absorbance of the resulting solution (total volume = 3.0 mL) was measured at 532 nm. The inhibition ratio of the extract (%) was calculated using the following formula:Inhibition ratio (%) = (*A*_control 532 nm_ − *A*_sample 532 nm_/*A*_control 532 nm_) × 100

### *In vivo* antioxidant properties

#### Acute toxicity studies: lethal dose 50 (LD_50_)

Acute toxicity study was performed for *Mc*EO in male wistar rats as per OECD guidelines. A single dose of the oil was administered orally to each animal. The animals were fasted overnight and provided only water, after which the *Mc*EO were treated with graded doses of *Mc*EO (100 mg, 200 mg, 400 mg kg^−1^ and 5000 mg kg^−1^, i.g.) and observed for 14 days to assess the acute oral toxicity of *Mc*EO*.* The animals were observed individually during the first 30 min and thereafter 24 hourly for a period of 14 days.^18^

##### Animal

Wistar rats weighing 200 to 220 g were obtained from the Central Pharmacy of Tunisia. They were kept in cages in a breeding farm at a temperature of 21 ± 1 °C with alternating periods of 14 h darkness and 10 h illumination, with a relative humidity around 40%. All rats had free access to drinking water and diet. The pelleted diet for rats was 15% protein and supplied by the Industrial Society of Concentrate (SICO, Sfax, Tunisia). The experimental protocol was approved by the ethical Committee of the Faculty of Sciences of Sfax with ethics approval number 1204. All the experimental procedures were carried out in accordance with international guidelines for care and use of living animals in scientific investigations (Council of European Communities).

##### Experimental design

Rats were randomly assigned to four groups of eights animals each. Animals of the first group receiving distilled water and standard laboratory diet, served as controls (C). Second group (CCl_4_), hepatotoxicity model, was given a single dose of CCl_4_ (1 mL kg^−1^ in 1% olive oil. ip)^19^ on the 14^th^ day. Animals of the third group (*Mc*EO) were given during 15 days a daily i.p. injection of *Mc*EO at 250 mL kg^−1^ b.w^19^ and distilled water as sole beverage. The fourth group (*Mc*EO + CCl_4_) was pretreated with *Mc*EO and intoxicated with CCl_4_ on the 14^th^ day. During the 2 weeks of experimental period, all animals survived.

##### Organ sampling

At the end of the experiment period (15 days), 24 h after the administration of CCl_4_, control and treated rats were anesthetized with chloral hydrate by intra-abdominal injection. The body weight of rats was recorded and blood samples were collected in heparin tubes by brachial artery. At the end of the experimental period, the animals of different groups were killed by cervical decapitation to avoid animal stress. Plasma samples were obtained from blood after centrifugation at 2500*g* for 15 min, to estimate some selective serum biochemical parameters. They were kept at −20 °C until analysis. All samples were analyzed in triplicate.

The livers were collected, cleaned and weighed. Some samples were homogenized (1 : 2, w/v) in 50 mmol L^−1^ Tris buffer (pH 7.4) containing 150 mmol L^−1^ of NaCl using an ultra-Turrax device. The homogenates were centrifuged at 5000 g for 25 min at 4 °C and aliquots of supernatant were kept at −20 °C until analyses. In parallel, portions of liver were immediately fixed into Bouin solution (saturated picric acid added with 37–40% formaldehyde and glacial acetic acid, 75 : 25 : 5 v/v) for histological studies.^20^

### Biochemical assay

#### Protein quantification

Protein content was evaluated as described by Lowry *et al.* (1951)^21^ using bovine serum albumin (BSA) as standard.

#### Measurement of thiobarbituric acid reactive substance (TBARS)

The formation of lipid peroxides was measured in the liver. The formation of MDA, a product of fatty acid peroxidation was measured spectrophotometrically at 532 nm using a thiobarbituric acid reactive substance (TBARS), essentially by the method of Yagi (1976).^22^ Results are expressed in nmoles of MDA formed/mg protein.

#### Measurement of protein carbonyl (PCO)

Protein carbonyl content in liver tissue was measured using the DNPH method by Reznick and Packer (1994).^23^ In brief, 100 mL of kidney extract supernatant were placed in glass tubes. Then, 500 mL of 10 mM 2,4-dinitrophenyl hydrazine (DNPH) in 2 N HCl were added. Tubes were incubated for 1 h at room temperature. Samples were vortexed every 15 min. Then, 500 mL of TCA (20%) were added and the tubes were left on ice for 5 min followed by centrifugation for 10 min. Protein precipitates were collected. The pellet was then washed twice with ethanol–ethyl acetate (v/v). The final precipitate was dissolved in 600 mL 6 M guanidine hydrochloride solution and incubated for 15 min at 37 °C. The absorbance of the sample was measured at 370 nm. The carbonyl content was calculated based on the molar extinction coefficient of DNPH (*£* = 2.2 × 10^4^ cm M^−1^) and the results were expressed as nmol per mg of protein.

#### Measurement of superoxide dismutase (SOD) activity

SOD activity was estimated according to Beyer and Fridovich (1987).^24^ Developed blue color in the reaction mixture was measured at 560 nm. Units of SOD activity were expressed as the amount of enzyme required to inhibit the reduction of nitroblue tetrazolium (NBT) by 50%, and the activity was expressed as units per mg of protein.

#### Measurement of catalase (CAT) activity

Catalase activity was assayed by H_2_O_2_ consumption, following Aebi (1984)^25^ method and modified by Pieper *et al.*^26^ Briefly, ethanol was added (1 : 100, v/v) to the supernatants and incubated for 30 min in an ice bath. 1% Triton X-100 (1 : 10, v/v) (Sigma Chemicals Corporation, MO) was then added to the homogenates. This solution was placed in an ice bath for an additional 15 min. 500 mL of this solution were placed into a glass cuvette and 250 mL of 30 mM H_2_O_2_ (Sigma Chemicals Corporation, MO) in phosphate buffer (50 mM, pH 7) was then added to start the reaction. The absorbency was monitored at 240 nm every 15 seconds for 45 seconds. Catalase activity was expressed in mmol H_2_O_2_ min mg^−1^ protein. An enzyme unit was defined as the amount of enzyme that catalyzes the release of one mmol of H_2_O_2_ per min. Specific activity was calculated in terms of units per mg of protein. The assay was performed at 25 °C.

#### Measurement of glutathione peroxidase (GPx) activity

Glutathione-peroxidase (GPx) activity was measured according to the method of Floke and Gunzler (1984).^27^ The enzyme activity was expressed in nmoles of GSH oxidized per minper mg of protein.

### Histopathological examination

After fixation in Bouin solution, pieces of fixed tissue were embedded into paraffin, cut into 5 μm slices and colored with hematoxylin–eosin to examine tissue constitution.^28^ Six slices were prepared from each liver. All sections were evaluated semi-quantitatively for the degree of liver injury. The steatohepatitis calculation system was applied to evaluate necrosis, inflammation, and ballooning.^29^

### Statistical analysis

All values are expressed as mean ± SE for continues variables or as median with inter quartile range [25%, 75%] where appropriate. The results were analyzed by One-Way Analysis of Variance (ANOVA) followed by Tukey test for multiple comparisons using SPSS for Windows (version. 12) or ANOVA-on-ranks with Dunn's correction. Differences were considered significant at *p* < 0.05.

## Results

### Chemical composition of the essential oil

**Fig. 1:**
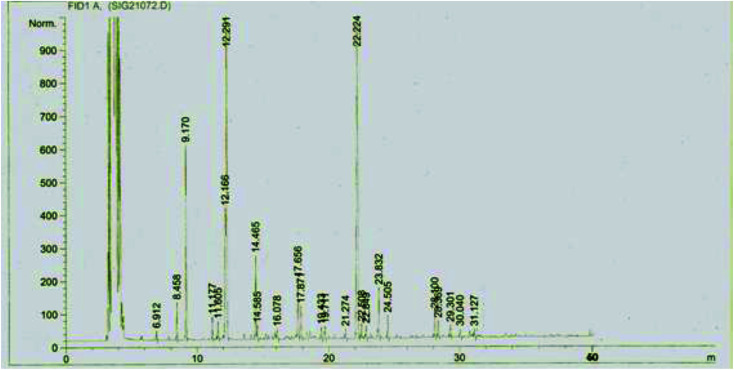
Chromatogram GC/MS of *Mc*EO.

**Fig. 2:**
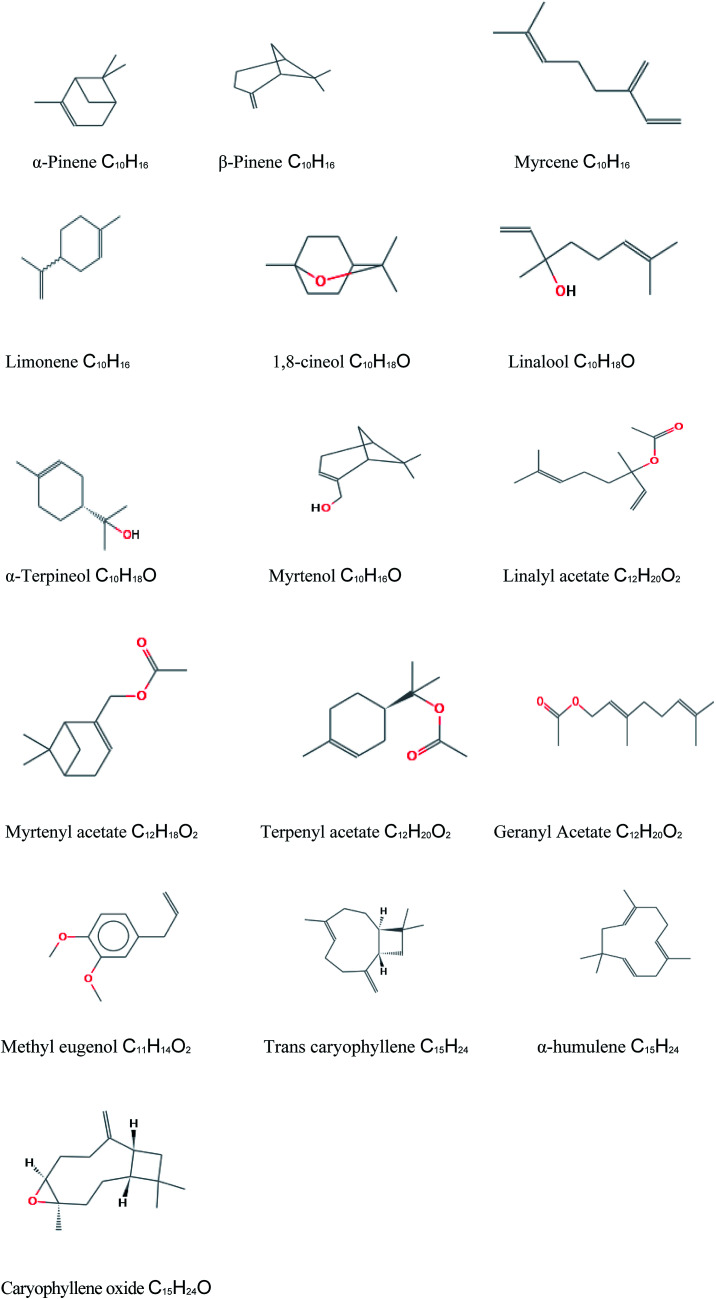
Structures of the different components detected in *Mc*EO.

The composition of *Mc*EO was assessed using GC-MS analysis (Fig. 1) and the details of volatile compound identification are presented in Table 1. The structures of *Mc*EO components of are given in Fig. 2. The hydrodistillation of *Mc*EO gave a yield of 2.8% (v/w) and 17 total components were identified, accounting for 97.86% of the whole oil. These components belong to two classes: hydrocarbon monoterpenes and oxygenated monoterpenes (Table 1, Fig. 1). Our oil was characterized by a high percentage of monoterpenes and especially hydrocarbon ones that constitute the predominant class (50.11%). Furthermore, the hydrocarbon monoterpenes were represented by α-pinene (35.20%) and limonene (8.94%). Other components were found such as 1,8-cineole (17%), linalool (6.17%), terpenyl acetate (4.30%), geranyl acetate (4.42%), methyl eugenol (6.98%) *trans*-caryophyllene (4.04%) and caryophyllene oxide (2.49%).

**Table 1:** Chemical composition of *Myrtus communis* flower essential oil (*Mc*EO) extracted by hydrodistillation

No.	Components^a^	*R*_t_ (min)^b^	KI^c^	%^d^
1	α-Pinene	9.18	939	35.20
2	β-Pinene	10.45	980	0.24
3	Myrcene	10.80	991	1.21
4	Limonene	12.12	1030	8.94
6	1,8-Cineol	12.26	1033	17.00
7	Linalool	14.45	1078	6.17
8	α-Terpineol	17.64	1090	3.86
9	Myrtenol	17.88	1194	0.42
10	Acetate linalyl	19.73	1257	0.85
11	Myrtenyl acetate	22.10	1325	1.26
12	Terpenyl acetate	22.80	1355	4.30
13	Acetate geranyl	23.80	1385	4.42
14	Methyl eugenol	24.48	1406	6.98
15	Trans caryophyllène	25.20	1415	4.04
16	α-Humulene	26.20	1460	0.48
17	Caryophyllene oxyde	30.05	1580	2.49
	Monoterpene hydrocarbon	50.11		
	Oxygenated monoterpenes	47.75		
	**Total %**	97.86		

a Identification of components based on GC-MS Wiley 7.0 version library and National Institute of Standards and Technology 05 MS (NIST) library data.

b *R*_t_: retention time.

c KI: Kovats Indices on HP-5MS Capillary Column in reference to C_10_–C_22_*n*-alkanes injected in the same conditions.

d %: percentages are the means of two runs and were obtained from electronic integration measurements using a selective mass detector.

### *In vitro* antioxidant properties

#### Antioxidant capacities of *Mc*EO

**Fig. 3:**
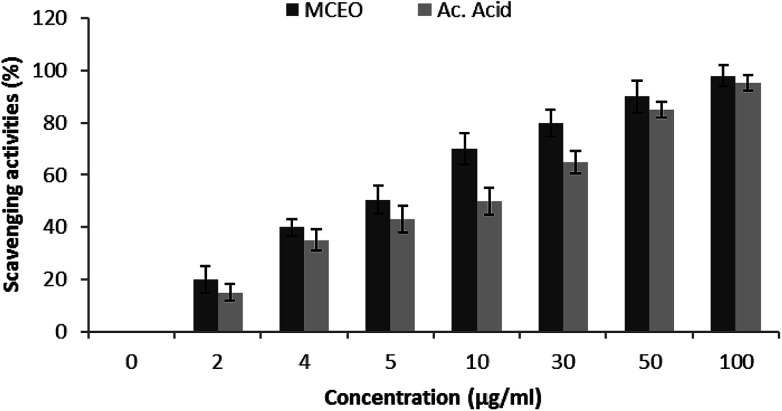
Scavenger effect of *Mc*EO at different concentrations, 0, 2, 4, 5, 10, 30, 50 and 100 μg mL^−1^, on the stable 1,1-diphenyl-2-picrylhydrazyl radical (DPPH). Results are expressed as percentage decrement of absorbance at 517 nm with respect to control. Ascorbic acid was used as a standard. Each value represents the mean ± standard deviation (*n* = 3).

**Fig. 4:**
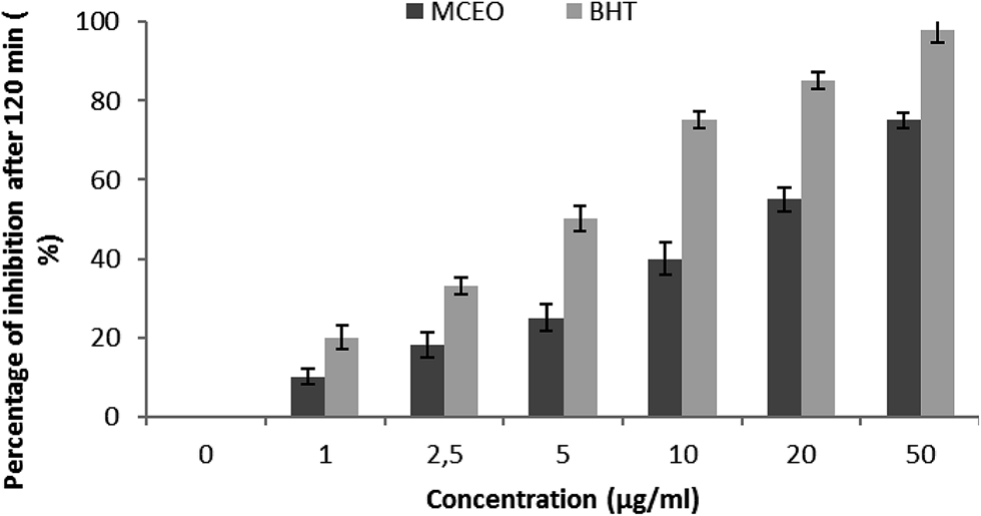
Antioxidant activities of *Mc*EO at different concentrations, 0, 1, 2.5, 5, 10, 20 and 50 μg mL^−1^ measured by β-carotene bleaching method. BHT was used as standard. Values are means ± standard deviation (*n* = 3).

The antioxidant potential of *Mc*EO was evaluated using DPPH radical scavenging method by comparing it with the activity of the ascorbic acid used as reference. The result of *Mc*EO DPPH free radical-scavenging ability is shown in Fig. 3 and compared with ascorbic acid. As shown in Fig. 3, the DPPH radical scavenging increased from 20% to 90.02%, when the concentration of the EO increased from 2 to 50 μg mL^−1^. The half maximal inhibitory concentration (IC_50_) of the EO and ascorbic acid were of 7.5 and 8 μg mL^−1^, respectively. According to these results, the *Mc*EO has a strong radical scavenging activity. The inhibitory effect of the *Mc*EO tested in different concentrations on lipid peroxidation was determined by the β-carotene/linoleic acid bleaching test. Fig. 4 showed a different degree of the linoleic acid oxidation and subsequently the β-carotene bleaching after the addition of the *Mc*EO and the BHT used as positive control at different concentrations. This antioxidant activity was dose-dependent as found in the DPPH test. Overall results were better than those provided by the radical-scavenging activity (Fig. 3 and 4).

**Fig. 5:**
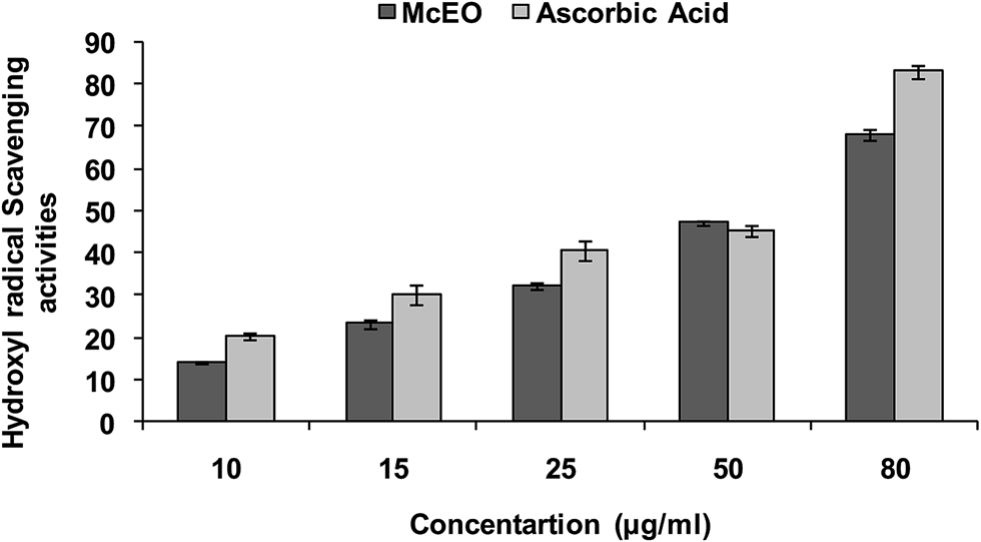
Percent scavenging activity for site-specific hydroxyl radicals in deoxyribose degradation assay from *M. communis* (*Mc*EO). Data represent the means ± SD (*n* = 3).

Hydroxyl radical (OH˙) could easily cross the cell membranes, and could readily react with most biomolecules including carbohydrates, proteins, lipids, and DNA in cells, causing tissue damage or cell death. Thus, removing ·OH was important for the protection of living systems. As shown in Fig. 5, the inhibition percentage of hydroxyl radical scavenging were observed as 14.02 (±0.7), 23.28 (±2.33), 32.27 (±2.5), 47.20 (±1.3), 68.04 (±1.5) μg mL^−1^ whereas for the standard ascorbic acid, it was found to be 20.25 (±0.3), 30.20 (±1.2), 40.66 (±0.7), 45.23 (±0.6) and 83 (±1.3) respectively. The IC_50_ value calculated for the *Mc*EO was of 47.20 ± 1.3 μg mL^−1^ against a value of 45.23 ± 1.3 μg mL^−1^ for ascorbic acid used as standard.

### Acute toxicity studies

No mortality was observed up to a dose level of 5000 mg kg^−1^ BW. Physically, the rats appeared normal and no signs of changes were observed in their skins, furs and eyes. Tremor, sleep and behavior patterns were similar to the normal group. Their food intakes were normal and neither diarrhea nor vomiting was noticed. Moreover, dissection results showed that there was no damage in liver.

### Effects of *Mc*EO on the marker enzymes status, ALP and LDH of liver function

**Fig. 6:**
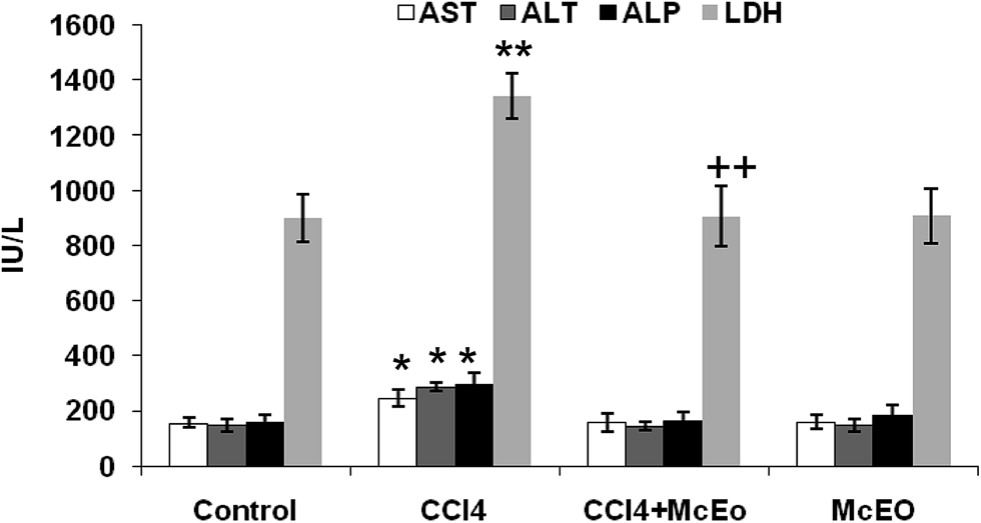
Plasma levels of bio-indices of liver functions in adult rats treated with CCl_4_ alone or concomitantly with *Mc*EO for 15 days. C: control; CCl_4_: carbon tetrachloride; (CCl_4_ + *Mc*EO): rat pre-treated with *Mc*EO and intoxicated with CCl_4_ at 14 day. AST, aspartate aminotransferase; ALT, alanine aminotransferase; ALP, alkaline phosphatases; LDH, lactate deshydrogenase. Values are expressed as mean ± SE of eights animals in each group. One-way ANOVA followed by Fisher's protected least significant difference (FLSD) as a *post hoc* test for comparison between groups: comparison between CCl_4_ and control groups: *P < 0.05; **P < 0.01. Comparison between CCl_4_ + *Mc*EO and CCl_4_ groups: ^++^*P* < 0.01.

Fig. 6 showed the plasma hepatic enzymes' levels, ALP and LDH of control and experimented rat. The administration of the *Mc*EO reestablished the CCl_4_ induced hepatic enzymes activities increase, in significant manner (*P* < 0.05). For example, AST activity was reduced in *Mc*EO pre-treated rats (valeurs) in comparison to those receiving only CCl_4_ (valeurs). Treatment with the *Mc*EO alone gave comparable enzymatic levels to sham group (example de valeurs) (Fig. 6).

### Effect of *Mc*EO on lipid profile

**Fig. 7:**
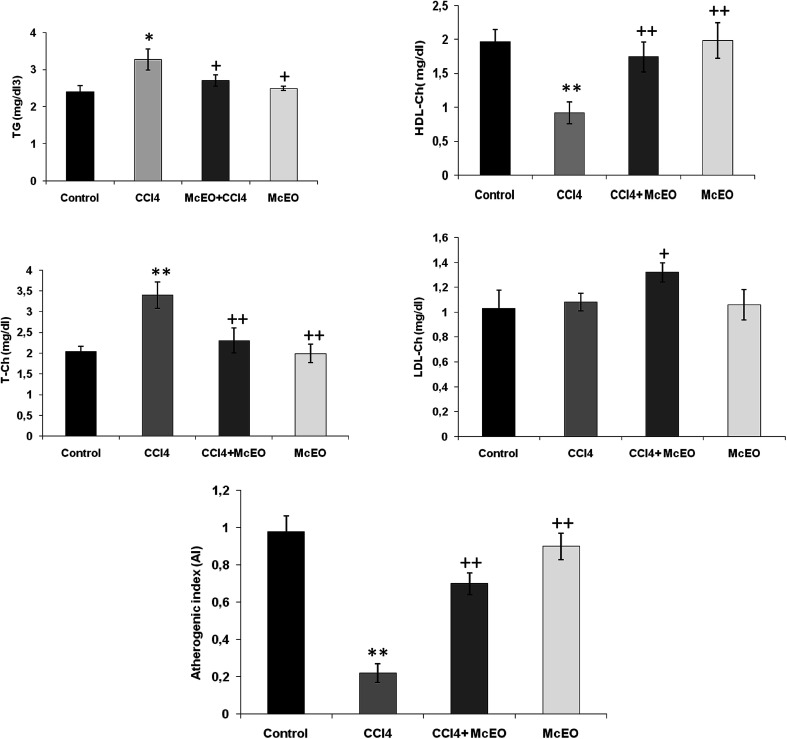
Lipids profile in serum of bio-indices of liver functions in adult rats treated with CCl_4_ alone or concomitantly with *Mc*EO for 15 days. C: control; CCl_4_: carbon tetrachloride; (CCl_4_ + *Mc*EO): rat pre-treated with *Mc*EO and intoxicated with CCl_4_ at 14 day; TG, triglycerides; T-Ch, total cholesterol; HDL-Ch, high density lipoproteins of cholesterol; LDL-Ch, low density lipoproteins of cholesterol, atherogenic index (AI) = (T-Ch − HDL-Ch)/HDL-Ch. Values are expressed as means ± SE of eights animals in each group. One-way ANOVA followed by Fisher's protected least significant difference (FLSD) as a *post hoc* test for comparison between groups: comparison between CCl_4_ and control groups: *P < 0.05; **P < 0.01. Comparison between CCl_4_ + *Mc*EO and CCl_4_ groups: ^+^*P* < 0.05, ^++^*P* < 0.01.

According to Fig. 7, in CCl_4_ intoxicated rats, the serum lipid classes (T-Ch, TG and LDL-Ch) increased (+33.9%, +84.9% and +118%, respectively). On the contrary, HDL-Ch level was significantly reduced (−52.8%, *P* < 0.001) in comparison to the normal control rats after 2 weeks of treatment with *Mc*EO alone. Pre-treated animals with *Mc*EO before CCl_4_ induced toxicity markedly reversed the serum lipid profile (TG, T-Ch and LDL-Ch levels) compared to the positive group. Furthermore, HDL-Ch level showed a significant increase (+88.2%, *P* < 0.001) as compared to CCl_4_ group.

In addition, we noted a clear decrease in HTR (%) and a corresponding increase in AI, T-Ch/HDL-Ch and LDL-Ch/HDL-Ch ratio in toxic state. These parameters were improved after 2 weeks of pre-treatment by the effect of EACA to a striking amount when compared to the toxic control (*P* < 0.001).

### *In vivo* antioxidative effect of *Mc*EO

Our findings revealed that *Mc*EO prevents the CCl_4_ induced hepatic oxidative stress misbalance. Table 2 summarizes the measured hepatic ROS balance parameters. It shows a significant decrease of the level of lipid peroxidation, as evaluated by TBARS, in *Mc*EO pretreated rats (valeurs) in comparison to non treated CCl_4_ intoxicated rats (+43.2%).

**Table 2:** Effect of CCl_4_ treatment and *Mc*EO supplementation on oxidative status and antioxidant system activity in adult rats/for 15 days^a^

Treatments	*n* = 8				
	^1^TBARS	^2^PCO	^3^SOD	^4^CAT	^5^GPx
Control	21.00 ± 3.15	67.00 ± 1.45	20.00 ± 1.15	42.50 ± 1.45	9.34 ± 0.22
CCl_4_	30.50 ± 2.30**	92.20 ± 2.49**	11.00 ± 0.21***	29.00 ± 1.20**	4.87 ± 0.13***
CCl_4_ + *Mc*EO	24.00 ± 2.10^+^	56.00 ± 2.34^++^	20.00 ± 1.10^+++^	42.29 ± 1.20^+++^	8.76 ± 0.26^+++^
*Mc*EO	21.00 ± 3.59^++^	60.00 ± 2.99^++^	20.27 ± 0.35^++^	41.96 ± 1.19^++^	8.94 ± 0.15 ^++^

a C: control; CCl_4_: carbon tetrachloride; (CCl_4_ + *Mc*EO): rat pre-treated with ethyl acetate fraction from extract *C. aurantium* and intoxicated with CCl_4_ at 14 day. ^1^TBARS, thiobarbituric acid reactive substances (nmol mg^−1^ protein). ^2^PCO, protein carbonyl (nmol mg^−1^ protein). ^3^SOD, superoxide dismutase (U SOD per mg protein). ^4^CAT, catalase (mmol mg^−1^ protein). ^5^GPx, glutathione peroxidase (nmol mg^−1^ protein). Values are expressed as means ± SE of eights animals in each group. One-way ANOVA followed by Fisher's protected least significant difference (FLSD) as a *post hoc* test for comparison between groups: comparison between CCl_4_ and control groups: *P < 0.05; **P < 0.01; ***P < 0.001. Comparison between CCl_4_ + *Mc*EO and CCl_4_ groups: ^+^*P* < 0.05; ^++^*P* < 0.01; ^+++^*P* < 0.001.

Similarly, a remarkable leveling-down of the oxidized proteins in (*Mc*EO + CCl_4_)-group (valeur) when compared to CCl_4_ one (+37.5%), while a significant decrease (*P* < 0.001) in the activities of enzymatic antioxidants (SOD, CAT and GPx) in the CCl_4_-treated rats compared with controls; the pre-administration of *Mc*EO significantly improved the antioxidant status in the liver tissue compared to CCl_4_-treated group. Moreover, the *Mc*EO group showed no noticeable variation in the activities of these enzymes compared with the control one (Table 2).

### Histopathological studies

**Fig. 8:**
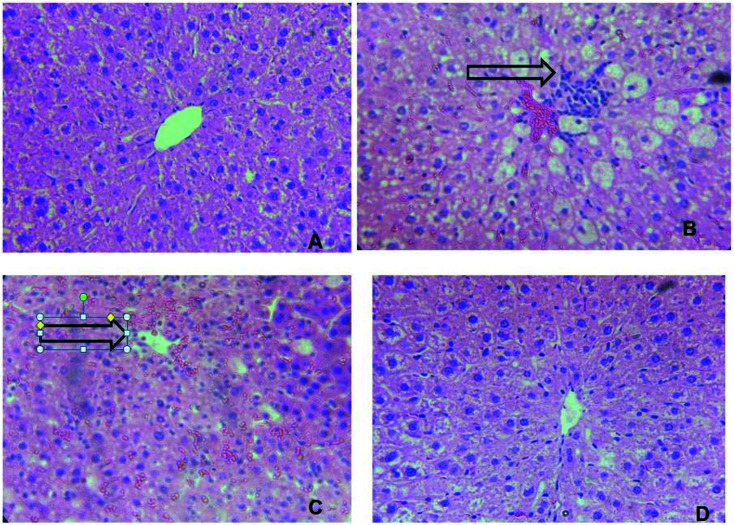
Effect of *Mc*EO histological morphology on fibrosis rat liver with Masson staining (*100). (A) Control group; (B) *Mc*EO group; (C) CCl_4_ treated group; (D) CCl_4_ and + *Mc*EO group.

As shown in Fig. 8, the hepatic tissue was normal in the control group (Fig. 3a). The treatment with CCl_4_ caused hepatocytes' vacuolization, enlargement of nuclei and ballooning degenerations, associated with neutrophilic infiltration and a significant congestion of the sinusoids (coagulative necrosis). The lymphocytic infiltration in the portal triads and sinusoids is frequently observed in the case of swelling of the liver cells (Fig. 8b). However, these hepatic lesions induced by CCl_4_ were considerably reduced by the administration of *Mc*EO (Fig. 8d) in the CCl_4_ + *Mc*EO groups. The histological pattern was almost normal in rats treated with *Mc*EO (Fig. 8c)

## Discussion

Our study was carried out to explore the protective effect of *Mc*EO on CCL_4_-induced hepatotoxicity in rats in rats. Our study is considered as a first record of the chemical composition of Tunisian *M. communis* flower EO. Tuberoso *et al.* (2006)^30^ and Djenane *et al.* (2011)^31^ reported that 1,8-cineole and α-pinene were the main constituents of *M. communis* EOs, which is in good agreement with our results. Tuberoso *et al.* (2006)^30^ reported that the chemical composition of *Myrtus* species exhibited small qualitative differences. Nevertheless, large variations depending on the origin of the samples were observed in the concentration of the main constituents. Generally, the amount of α-pinene was 30% for all samples except for one whose content was two-fold higher (59.50%). Limonene percentage ranged from 5.20 to 29.80%; that of 1,8-cineole ranged from 15.90 to 41.70% whereas the amount of linalool varied from 0.20 to 16.70%. α-Terpineol and geranyl acetate amounts ranged respectively from 1.30 to 4.80% and 0.40 to 7.20%. However, the chemical composition of this oil was different of those reported for EOs isolated from *M. communis* leaves and berries growing wild all around the Mediterranean basin.^32^ Other studies showed that, among the constituents of the *M. communis* leaf and berry EOs, myrtenol, myrtenal and myrtenyl acetate presented the major compounds.^33^ The chemical composition also depends on season or vegetative period of plant.^34^ According to these factors, plant biosynthetic pathways can influence the relative proportions of EO components.

In the present study, the 50% inhibition concentration of our EO for scavenging the hydroxyl activity was of 47.20 ± 1.3 μg mL^−1^ whereas that of for the standard antioxidant was equal to 45.23 ± 0.6 μg mL^−1^. This clearly depict that the *Mc*EO the ability to scavenge the hydroxyl radical produced even though the activity is somewhat moderate when compared with the ascorbic acid which has shown strong antioxidant activity. Additionally, regarding the inhibition of lipid peroxidation by addition of the *Mc*EO could be used to improve the quality and stability of food products. The *Mc*EO was able to quench peroxide radicals and to block the peroxidation chain reaction.

It is important to note that the antioxidant activities of the studied EOs are due essentially to the abundance of hydrocarbon monoterpenes hand may be to the synergy between the overall chemical constituents.^35^ The *Mc*EO and their active components, showed excellent antioxidant capacities compared with the standard antioxidant. It seems to be a general trend that EOs which contain monoterpene hydrocarbons, oxygenated monoterpenes and/or sesquiterpenes; have greater antioxidative properties.^14^ These activities may be attributed to the presence of 1,8-cineole, α-pinene, β-pinene and limonene.^36^ For example, the high scavenging activity reported for two *Rosmarinus officinalis* L. varieties could be explained partially by the high amounts of camphor, linalyl acetate and α-thujene recorded in these oils.^14^ However, it is difficult to attribute the antioxidant effect of a total essential oil to one or few active compounds. Both minor and major compounds should make a significant contribution to the oil's activity.^14,36^ The *Mc*EO can be used as an easily accessible source of natural antioxidants.

Numerous studies clearly demonstrated the importance of medicinal plants in the treatment of oxidative stress-induced cell death.^37^ The present study was undertaken to study the possible hepatoprotective role of the *Mc*EO in CCl_4_ induced liver toxicity rat model.

CCl_4_ is a chemical hepatotoxin known for inducing in animal model features similar to those of acute hepatitis in human. CCl_4_ is metabolized by cytochrome P450 system and converted to trichloromethyl and trichloromethyl peroxy radicals^38^ which initiates peroxidation of polyunsaturated fatty acid (PUFA) of cell membranes with secondary damage, severe enzymatic disturbances, and increases MDA production.^39^

Liver is one of the main organs involved in the metabolism of drugs and toxic chemicals. It is the first target organ for almost all chemicals.^40^ Most of xenobiotics enter the body through gastrointestinal tract and after absorption enter the liver through portal vein. CCl_4_ is widely used for induction of liver damage in experimental animals^41^ that mimic human hepatic toxicity.^42^ The liver injury is a major health problem which may develop into several liver diseases. It is mainly attributed to the reactive oxygen species and free radicals generated during its metabolism.^2,43^ In the current study, CCl_4_ injected at day 14 successfully induced fulminant characterized by decreased body weight and non-lethal hepatotoxic phenomena in rat, which was consistent with previous reports.^44^ Moreover, Previous studies have already reported that the xenobiotic cause different damages at the hepatic levels in rats under different experimental conditions.^45^ AST, ALT, ALP and LDH are important enzymes linking carbohydrate and amino acid metabolisms. These enzymes are often used in the evaluation of hepatic disorders. In fact, an increase in their activities reflects acute liver damage and inflammatory hepatocellular disorders.^12^ Hence, our results revealed that the injection of CCl_4_ to rats caused a significant increase in AST, ALT, ALP and LDH activities at day 14 of treatment. This is in agreement with previous reports,^43,46^ suggesting that an extensive liver injury was occasioned by CCl_4_ due to changes in their functional transition. These changes cause membrane permeability, and leads to the leakage of enzymes into extracellular space.

The pretreatment with the *Mc*EO before the injection of CCl_4_ significantly reduced the elevation of serum level of ALT, AST ALP and LDH. This result showed that the *Mc*EO has the ability to lower the increased serum enzymes levels resulting from the administration of CCl_4_ alone; indicating structural and functional integrity of hepatic parenchyma cells.

As one of the principal causes of CCl_4_-induced liver injury, lipid peroxidation is mediated by the free-radical derivatives of CCl_4_. The antioxidant activity and the inhibition of free radical generation are important in terms of protecting the liver from CCl_4_-induced damage.^47^ The body has an effective defense mechanism to prevent and neutralize the free radical-induced damage. This is accomplished by a set of endogenous antioxidant enzymes which are able to detoxify free radicals by converting them back to more stable molecules within the cell, to be used or disposed accordingly.^48^

Lipids play an important role in hepatic disease incidence. This study has also revealed that the CCl_4_ treatment induced perturbation of lipid metabolism of (triglyceride and cholesterol levels). In fact, CCl_4_ caused a significant (*P* < 0.001) increase in the levels of TG, Ch and LDL-Ch with a concomitant decrease in HDL-Ch level. The increase in cholesterol levels might be due to the increased esterification of FA, inhibition of FA β-oxidation, and decreased excretion of cellular lipids.^49^ CCl_4_ stimulates the transfer of acetate into liver cells and leads to an increase in cholesterol synthesis. It also increases the synthesis of FA and TG from acetate and stimulates lipid esterification.^50^ Moreover, the findings of Kamalakkannan *et al.*^50^ indicated that CCl_4_ inhibits the synthesis of apo-lipoprotein thus reducing the synthesis of lipoproteins. The pre-treatment with the *Mc*EO restored the lipid parameters indicating its ability to regenerate or protect hepatic cell membrane integrity (decreased cholesterol, triglyceride, and LDL levels and increased HDL level). Among the antioxidant compounds, phenolic compounds are the most efficient in terms of lipid peroxidation inhibition.^50,51^

To understand the mechanisms of the *Mc*EO protective effect against acute CCl_4_-induced liver injury, we evaluated the activities of antioxidant enzymes (CAT, SOD and GPx), as well as the level of MDA in rat liver. The results showed that the levels of CAT, SOD and GPx were significantly lower and the level of MDA was significantly higher in CCl_4_ alone-treated rats as compared with those of control group. The pre-treatment with the *Mc*EO in CCl_4_-treated rats exhibited a significant decrease in TBARS level in the liver tissue pointing out an inhibitory role of the *Mc*EO against lipid peroxidation and, thereby, diminishing CCl_4_ induced hepatic damage. The prevention of lipid peroxidation could be attributed to the capability of the *Mc*EO to scavenge (ROS).^52^

CCl_4_-induced generation of peroxy and superoxide radicals results in the inactivation of catalase and SOD. These phenomena ultimately results in oxidative stress and hepatocyte injuries. The reduced activity of these enzymes could be due to an enhanced lipid peroxidation or an inactivation of the antioxidant enzymes.^19^ Our results indicated that pretreatment with the *Mc*EO caused an increase in the activity of antioxidant enzymes. The antioxidant enzyme system plays an important role in the defense of cells against oxidative insults. The study examined the ameliorating effect of the *Mc*EO, on oxidative stress induced by CCl_4_.

These results suggest that the *Mc*EO reduced the oxidative stress by preventing the generation of free radicals. The reduced oxidative stress and lipid peroxidation observed in the *Mc*EO treated animals may be attributed to the important role of EOs as antioxidants. This power may be attributed to their ability to decompose free radicals by quenching ROS and trapping radicals before reaching their cellular targets.^53^ The antioxidant activity of the *Mc*EO could also be assigned to the presence of hydrocarbon monoterpenes. Moreover, the measured antioxidant activities could be due to the synergistic effects of two or more compounds present in the oil.^54^ In this context, Kim *et al.* (2008),^55^ reported that most natural antioxidative compounds often act synergistically to produce a broad spectrum of antioxidative properties creating an effective defence system against free radicals.^56^

It is worthy noticing that the histological observations basically supported the results obtained from serum enzyme assays. The liver of CCl_4_-intoxicated rats showed massive fatty changes such necrosis and ballooning degenerations of hepatocytes. The histopathological observations of the liver rats pretreated with the *Mc*EO and subsequently given CCl_4_ showed a more or less normal architecture.

## Conclusion

In conclusion, it may be mentioned that the altered biochemical and oxidative stress profiles because of exposure to CCl_4_ is reversed by *Mc*EO. The contents of *Mc*EO not only protect the integrity of plasma membrane but, at the same time, increased the regenerative and reparative capacity of the liver. These results suggest that the compound present in *Mc*EO has hepatoprotective effects against CCl_4_ induced oxidative stress in rats as evidenced by lowering TBARS level in the liver tissue, and liver marker enzymes in the serum. Therefore, *Mc*EO has a antioxidative effect against the toxicity induced by CCl_4_. For all that, as our investigations stand at present, it turns out that complementary studies *in vitro* and *in vivo* will be necessary to test effect of each compound in more detail.

## Conflicts of interest

The authors declare that there are no conflicts of interest.

## Abbreviations

ALP: Alkaline phosphatases
ALT: Alanine aminotransferase
AST: Aspartate aminotransferase
AI: Atherogenic index
BHT: Butylatedhydroxytoluene
b.w: Body weight
CAT: Catalase
DPPH: 1-Diphenyl-2-picrylhydrazyl
GPx: Glutathione peroxidase
HDL-Ch: High density lipoproteins of cholesterol
LD_50_: Lethal dose 50
LDL-Ch: Low density lipoproteins of cholesterol
IC_50_: Half maximal inhibitory concentration
LDH: Lactate deshydrogenase
*Mc*EO: *Myrtus communis* essential oil
MDA: Malondialdehyde
OSI: Organo-somatic index
PCO: Protein carbonyl
PUFA: Polyunsaturated fatty acid
ROS: Reactive oxygen species
SOD: Superoxide dismutase
TBA: Thiobarbituric acid
TBARS: Thiobarbituric acid reactive substances
TBS: Tris-buffer saline
T-Ch: Total cholesterol
TG: Triglycerides

## Supplementary Material
